# Basic Substances, a Sustainable Tool to Complement and Eventually Replace Synthetic Pesticides in the Management of Pre and Postharvest Diseases: Reviewed Instructions for Users

**DOI:** 10.3390/molecules27113484

**Published:** 2022-05-28

**Authors:** Gianfranco Romanazzi, Yann Orçonneau, Marwa Moumni, Yann Davillerd, Patrice André Marchand

**Affiliations:** 1 Department of Agricultural, Food and Environmental Sciences, Marche Polytechnic University, Via Brecce Bianche, 60131 Ancona, Italy; m.moumni@staff.univpm.it; 2 Institut Technique de l’Agriculture et de l’Alimentation Biologiques (ITAB), 149 rue de Bercy, 75012 Paris, France; y.orco17@gmail.com (Y.O.); yann.davillerd@itab.asso.fr (Y.D.); patrice.marchand@itab.asso.fr (P.A.M.)

**Keywords:** European Union, fungicide residues, plant protection, regulation EU 1107/2009

## Abstract

Synthetic pesticides are widely used to protect crops from pathogens and pests, especially for fruits and vegetables, and this may lead to the presence of residues on fresh produce. Improving the sustainability of agriculture and, at the same time, reducing the adverse effects of synthetic pesticides on human health requires effective alternatives that improve the productivity while maintaining the food quality and safety. Moreover, retailers increasingly request fresh produce with the amounts of pesticides largely below the official maximum residue levels. Basic substances are relatively novel compounds that can be used in plant protection without neurotoxic or immune-toxic effects and are still poorly known by phytosanitary consultants (plant doctors), researchers, growers, consumers, and decision makers. The focus of this review is to provide updated information about 24 basic substances currently approved in the EU and to summarize in a single document their properties and instructions for users. Most of these substances have a fungicidal activity (calcium hydroxide, chitosan, chitosan hydrochloride, *Equisetum arvense* L., hydrogen peroxide, lecithins, cow milk, mustard seed powder, *Salix* spp., sunflower oil, sodium chloride, sodium hydrogen carbonate, *Urtica* spp., vinegar, and whey). Considering the increasing requests from consumers of fruits and vegetables for high quality with no or a reduced amount of pesticide residues, basic substances can complement and, at times, replace the application of synthetic pesticides with benefits for users and for consumers. Large-scale trials are important to design the best dosage and strategies for the application of basic substances against pathogens and pests in different growing environments and contexts.

## 1. Introduction

The world population continues to grow and will reach 9.7 billion by 2050 [1]. For this, increasing food production is the primary objective of all countries. According to the latest estimates of the Food and Agriculture Organization of the United Nations [2], up to 40% of food crops worldwide are lost every year due to pests and plant diseases. Crop losses caused by plant disease alone cost the global economy $220 billion annually [3]. Crop protection is essential to reduce yield losses, improve food quality, and increase grower profitability. The application of plant protection products (PPPs) is the main way to protect crops against pathogens, pests, and weeds [4]. However, human, animal, and environmental risks associated with the use of chemical PPPs are a growing concern. All these concerns have encouraged the onset of research to develop alternative approaches to control plant diseases [5]. Reducing the use of pesticides being a major challenge in developed countries, European Union Member States are required to implement National Action Plans that set quantitative objectives, timetables, and indicators related to reducing the impact of pesticide use (Directive 2009/128/CE) [6,7]. The use of basic substances is approved in the European Union under Article 23 of EC Regulation No 1107/2009 and which are listed in Part C of the Annex of the Regulation (EC) No 540/2011 [8]. In the EU, Integrated Pest Management (IPM) has been mandatory since January 2014, and among the rules of the IPM is the reduction of the application of synthetic pesticides whenever possible [9]. For sustainable and qualitative food production, respectful of the need to produce in sufficient quantities, biocontrol has grown tremendously through the last few years [10]. The PPP EU Regulation (EC) 1107/2009 was established to ensure a level of protection of humans, animals, and the environment and, at the same time, to unify for the entire EU the rules on the placing on the market of plant protection products [11,12]. Basic substances are sources of interest for research as alternative to synthetic pesticides, since they are used in human medicine or as a food ingredient, so they have no residue concerns and then no maximum residue limit (MRL) and, usually, no preharvest interval [13,14]. The lack of MRL contributes to a better prevention of contamination in plant protection, a better control of the residues and a reduction of analytical problems, of decommissioning, and of market withdrawal [14]. Another benefit of basic substances, and perhaps the most important, is their very low ecologic impact. Basic substances are products that are used as ‘foodstuffs’, as defined in Article 2 of Regulation (EC) 178/2002 [15] cosmetic, and does not have an inherent capacity to cause endocrine-disrupting, neurotoxic or immunotoxic effects, but they are also plant protection means and not placed on the market as a plant protection product. Article 28 of Regulation (EC) No. 1107/2009 set the absence of marketing authorizations and usages allowance for basic substances. Regulation (EC) No. 1107/2009 introduced the new category of ‘basic substances’, which are defined by recital 18 as ‘certain substances which are not predominantly used as plant protection products may be of value for plant protection, but the economic interest of applying for approval may be limited. Therefore, specific provisions should ensure that such substances, as far as their risks are acceptable, may also be approved for plant protection use’. The properties of basic substances are described in Article 23 of the EU Regulation (EC) No 1107/2009 [11]. In 2021, the Euphresco project ‘BasicS’ contributed to demonstrate the effectiveness toward pests and pathogens of basic substances, with potential benefits for the farmers, the consumer, and the environment [16,17]. The basic substances have a positive impact on crop health when applied preventively. Certain basic substances, such as chitosan, stimulate the defense system of crops against several classes of pathogens, including fungi, viruses, bacteria, and phytoplasma [18]. According to the EU pesticides database, 24 basic substances were approved for use, 7 were withdrawn, 18 applications were not approved and 8 are still pending [19,20]. This review includes currently approved basic substances that have a protective potential and are a valuable addition to the range of measures and protection methods intended for use. Detailed information about basic substances and updates on new available compounds can be found at the page https://ec.europa.eu/food/plant/pesticides/eu-pesticides-database/active-substances (accessed on 23 May 2022). The standard-folder for approval of a basic substance, called ‘Basic Substance Application Template (BSAT)’, is based on the structure of the European Union evaluation report of an active substance that can be used for plant protection purposes. BSAT refers to all areas of risk assessment in the regulation of phytopharmaceutical product uses and shall be considered as a structured model to build a file collating all available information and enabling to demonstrate that the evaluated substance meets the eligibility criteria of a basic substance (SANCO 10,363 rev.10, 2021). Therefore, nowadays, a full deposit under International Uniform ChemicaL Information Database (IUCLID) software is mandatory since March 2021. Basic substances are submitted individually (Annex I inclusion dossier) at the first stage; then, later, an automatic inclusion was adopted for food/foodstuff basic substance from plant or animal origin [21,22]. Recently, an automatic consideration procedure (without any Annex I inclusion dossier) by Expert Group for Technical advice on Organic Production (EGTOP)/Directorate-General for the Agriculture and Rural Development (DGAgri) of positive ongoing basic substance approval (from Directorate-General Health and Food Safety—DGSanté to DGAgri) to generate an automatic EGTOP/DGAgri outcome for inclusion (or not). This provision bypasses the traditional route of substances in organic production in plant protection through dossiers submitted to Member States, but so far, no basic substance has been rejected by the Regulatory Committee of Organic Production (RCOP), and with the current procedure, are no longer studied than substances of mineral origin (or non-foods).

This review aimed to highlight the properties of approved basic substances, summarize, and provide this information for phytosanitary consultants, scientists, growers, stakeholders, companies, and consumers.

## 2. Results

**Figure 1:**
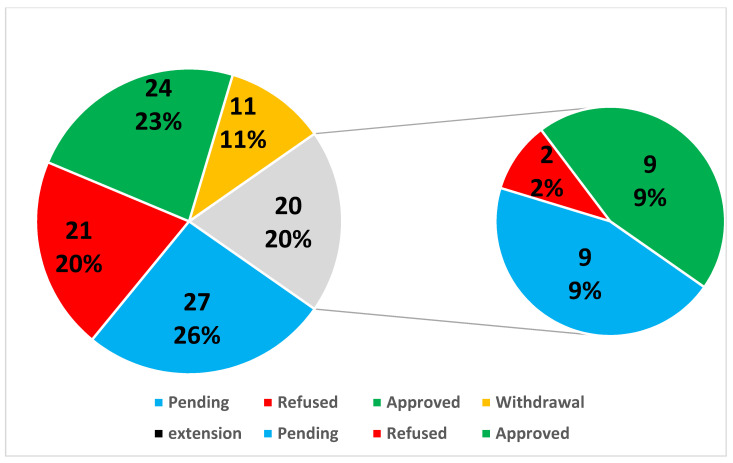
Total of the basic substance applications (BSA) and extensions presented by the results (%).

Out of the 86 basic substance application submitted to the European Commission until now, less than one-third have been approved (24) (Table 1 and Table 2), 19 have been refused, 6 have been withdrawn during their assessment (Table 3), 8 are currently being processed by the EC (Table 4 and Figure 1), and 2 already successfully submitted via IUCLID software (Ginger extract and *Capsicum frutescens*). 

Currently, 24 basic substances are approved, of which 21 are also approved in organic production; for example, talc was validated in 2021 following EGTOP PPP VII and is being currently voted on at RCOP [23] and clayed charcoal was submitted. Recently, voted chitosan does not seem to be acceptable directly in organic production as the basic substance from its microorganism’s origin, although in the context of food quality. Basic substances are approved by EU Regulations, so the application month, where reported in Table 1, is related to the Northern Hemisphere.

**Figure 2:**
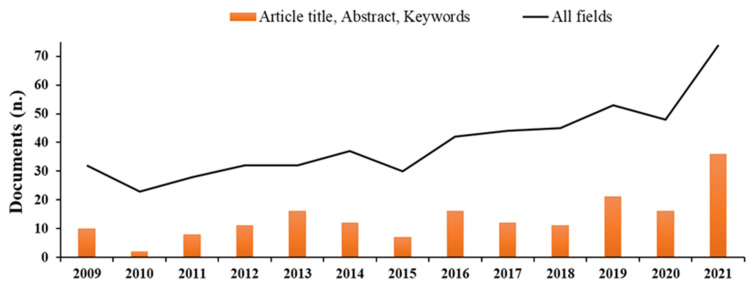
Number of documents available on Scopus through searches with keywords ‘basic substances’ in ‘Article title, Abstract, and Keywords’ (histograms) or in ‘All fields’ (linear) published over the last 10 years (Source: Scopus, https://www.scopus.com, accessed on 11 May 2022).

The scientific literature dealing with basic substances is relatively limited but increasing in recent years (Figure 2), and there is poor information about the effectiveness in field trials of basic substances toward pests and pathogens.

In the last decade, MRLs for pesticides with agricultural trade are becoming important. In the EU, there are increasing requirements from retailers to their suppliers to provide fruits and vegetables with an amount of pesticide residue below the MRLs (Table 5).

The substances tested during Casdar programs ‘4P’, ‘Carie’, ‘Sweet’, ‘HE, Ecophyto ‘Usage’ and some from projects have already been described (Marchand, 2016) (Table 6). New projects are ongoing to develop extensions of use, describe better efficacy through better positioning during the season or to investigate compatibility/incompatibility with other biocontrol agents (i.e., reduce copper and macro-organisms). This is the ongoing work for Coperreplace, ABAPIC (ITAB), Vitinnova (UNIVPM), and Euphresco BasicS (Euphresco Network).

## 3. Discussion

The use of pesticides, if not appropriate, may lead to problems like contamination of the water, potential damage to sensitive species (e.g., bees), contamination of final food products and water, with up to 90% of applied pesticides not reaching the target species, and, also, because of the development of resistant pathogens and pests [39]. A high number of PPPs were not reauthorized (or companies did not provide the dossier for the reregistration of products out of patent, due to high costs and uncertain benefits) and leaves a gap for several uses. It is important that authorities provide a good number of options to growers to protect their crops, since farmers cannot stand without PPPs for certain crops and uses, and there is an increasing need, because a lot of substance prohibition dates are fixed without substitution mean. Just as an example, this occurred with the fungicide mancozeb in January 2022 and a risk to occur in 2025 with copper, that is fundamental for plant protection in organic agriculture and a good support to prevent the appearing of resistant isolates in IPM. In France, the use of neonicotinoids, known as dangerous insecticides, is extended when there is no other way to preserve crops and productivity. With Farm to Fork Strategy of the European Green Deal, the European Commission is committed to reduce the use of the most dangerous synthetic pesticides of 50% and achieve at least 25% of the EU agricultural land under organic farming by 2030, although the decrease of synthetic pesticides is already ongoing. These trends, together with the implementation of sustainable development goals—SDGs by the United Nations—are demanding for new alternatives, such as basic substances, to tackle some of these issues. To achieve these goals, more research is needed to advance the design of better farming systems and the development of alternatives to synthetic pesticides and to copper formulations.

Three decades ago, the concept of MRLs was poorly known, while, in recent years, MRLs for pesticides arguably have become the first action growers should consider in their pest management decisions [40]. Trying to interpret consumer demands, retailers are increasingly required to reduce pesticide residues even more than the allowed thresholds (MRLs), which are defined considering a wide security factor (e.g., ×100) using the presence of pesticide residues as a factor of competition among companies. Requests from the retailers and consumer to reduce synthetic pesticide residues from fresh produce even more than the allowed threshold, such that the rules defined by the public administration have become more limiting for farmers in terms of the active ingredients allowed and MRLs [40,41]. The reduction of the presence of fungicide residues well beyond MRL may allow the pathogen to develop after harvest, resulting food loss and waste along the value chain. These developments have driven the search for alternative management strategies that are effective and not reliant just on conventional fungicide applications [5,42,43]. European regulation followed and carried this development with the introduction of new classes of phytosanitary products, in particular basic substances, but also new laws and simplification accompanied by the reduction of registration processes of low-risk substances, theoretically. Basic substances are approved for use in the EU and are products that are already sold for certain purposes, e.g., as a foodstuff or a cosmetic. Basic substances may be of major importance in biocontrol and several advantages can explain it. Basic substance regulatory application is simplified [44] and particularly reduced compared to other substances, therefore representing a lower cost to applicant (around 35-40 kEuro for approval of a basic substance and overall around 45 kEuro including approval for organic agriculture), thanks to the fact that these substances are already on the market for another purpose than plant protection, and safety is not an issue to be demonstrated. These substances are good alternatives available today and wide targets. Basic substances can be used in the crop protection as fungicide, bactericide, insecticide, etc., and most of them are allowed in organic production [18,45,46,47]. The basic substances are in order from 2014, when was the first approved application of *Equisetum arvense* L., chitosan hydrochloride, and sucrose until 2022, when a second chitosan formulation was approved. In some conditions basic substances were already at farm level, with a level of pest management not different than the standard. Just as example, chitosan hydrochloride was also applied in commercial conditions, in the field, and postharvest treatments, and several studies proved that it could have an effectiveness comparable to some commercial PPPs [42,48]. Basic substances, probably less efficient and practical to use than other active substances authorized as PPPs, are known and used by producers since decades as substitution means and have already demonstrated their effectiveness. Basic substances were the perfect tool to provide to producers as known, easy-to-use, less dangerous, and environmentally more respectful. Today, there is a consensus among a wide range of stakeholders that synthetic pesticide used need to be gradually reduced to a level that is effectively required to ensure crop production and that risks of pesticide application should be reduced as far as possible. Basic substances are good alternatives available today in our hands. The use of these substances needs to be integrated in vocational education, training, and technical advice to farmers. Further research around the world on the efficacy of basic substances may prove in the future that these substances can replace pesticides without reducing yields or increasing production costs. To develop the uses and the field trials we listed here the main usages of basic substances. However, rates included in the approval schedule may not produce a significant containment of diseases and pests in specific pathosystems. Just as example, the advised application rate of chitosan hydrochloride is between 100 and 800 g/ha, equal to a concentration ranging among 0.05 and 0.2% with 200–400 L/ha, while trials in commercial vineyards found a good effectiveness delivering the chitosan hydrochloride, with a concentration of at least 0.5% and with a volume of at least 500 L/ha [34,49]. For this reasons, large-scale trials are very important to demonstrate the effectiveness toward pathogens and pests in different environments and growing contexts, and a flexibility could be required in suggested dosages to avoid that applying basic substances at suggested rated can lead to a lack of or poor effectiveness and then the disaffection of users toward these innovative compounds, and this is in contrast with the requirements of finding solutions alternatives to the application of synthetic pesticides keeping the standard quality and quantity of the production, which is one of the drivers of the Farm-to=Fork Strategy of European Green Deal. Moreover, the diluent allowed for basic substance, up to now concretely restricted to water, may be another substance. In this case, vinegar has just been authorized for chitosan. Finally, increasing the demand from growers and competition among companies can lead to the reduction of costs of the treatments that, nowadays, are often higher than standard treatments.

## 4. Materials and Methods

### 4.1. Collection of Data

A systematic literature search from 2009 to 2021 was performed using the database of Scopus with the keywords ‘basic substance’ and ‘basic substances’. In the EU, several retailers request an amount of pesticide residue on fruit and vegetables below the legal limit (MRL), and data on some protocols were collected through companies and plant doctors.

### 4.2. Legislation

Basic substance criteria are defined by article 23 of Regulation (EC) No. 1107/2009, cited in introduction. By way of derogation from Article 4 of this regulation, a basic substance is approved when all relevant evaluations conducted in accordance with other Community legislation, governing other uses of this substance, showing that it has neither an immediate or delayed harmful effect on human or animal health nor any unacceptable influence on the environment. Active substances that could be defined as ‘foodstuff’ are intrinsically considered as basic substances, following Article 2 of Regulation (EC) No. 178/2002. Basic substances shall be approved in accordance with paragraphs 2–6 of regulation (EC) No. 1107/2009 and by way of derogation from Article 5, the approval shall be for an unlimited period. By way of derogation from Article 7 of Regulation (EC) No. 1107/2009, an application for approval of a basic substance can be made by a Member State or any interested party. At the end of the evaluation process, basic substances shall be listed separately in the Regulation referred to in Article 13(4). The Commission may review the approval of an active substance at any time. It may take into account the request of a Member State to review the approval. Article 28 of Regulation (EC) No. 1107/2009 set the absence of marketing authorizations and usages allowance for basic substances. However, no formal authorization is required as long as the product contains exclusively basic substances (see corresponding Review Report) [49,50].

### 4.3. Approval Process

**Figure 3:**
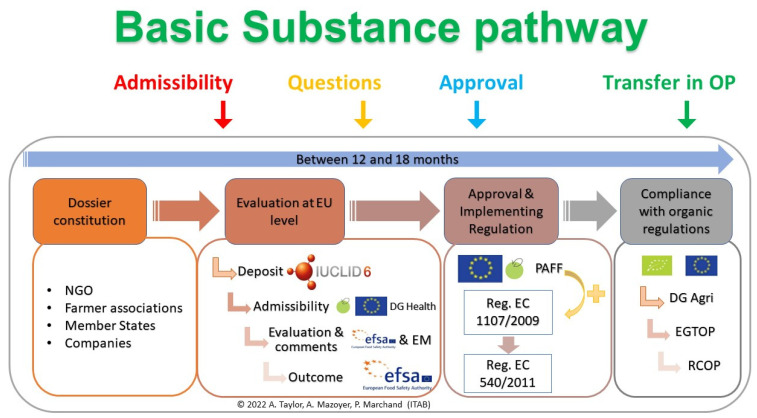
Approval process and timeline of a Basic Substance Application (BSA).

The approval process of a basic substance starts with a request for approval (Figure 3). The applicant estimates if the substance concerned fulfil all criteria of basic substances category and then complete the BSAT, in English, to obtain a Basic Substance Application. Several guidance documents, such as the official SANCO guide or the teaching guide from the ITAB, have been published to help applicants to build basic substance application correctly [50]. For the transmission of the basic substance application, once completed, the file should be sent to the DGSanté, representing the European Commission (EC). The Basic Substance Application can firstly be sent to national competent authorities for a preassessment and possibly a support. For example, in France, the Basic Substance Application can be sent to the Ministry of Agriculture (DGAl in France), who can ask for the National Authority’ opinion and then transfer the file to the EC. Upon receipt of the Basic Substance Application, EC implements the approval procedure detailed in Article 23 of Regulation (EC) No. 1107/2009. Admissibility may be pronounced at any time, directly or after questions from DGSanté. It constitutes the real start of the application (black line in Figure 3). The first stage is based on the Basic Substance Application evaluation by Member States and EFSA as scientific assistance leading to a request for corrections and questions. The request is sent to the applicant, and his answers shall be sent back within one month to the EFSA. For decision and approval, at the end of the basic substance application evaluation, EFSA will deliver its opinion, append a comment, and send the basic substance application to the DG Health within 3 months for the final vote of Member States in the PAFF committee (Figure 3). Approval, if accorded, is effective at the date of the publication of an implementing Regulation modifying Regulation (EU) No. 540/2011 [8].

The period of examination of the basic substance application is established in paragraph 1 of article 37 of Regulation (EC) No. 1107/2009. It is said: ‘The Member State examining the application shall decide within 12 months of receiving it whether the requirements for authorization are met. Where the Member State needs additional information, it shall set a period for the applicant to supply it. In that case, the 12-month period shall be extended by the additional period granted by the Member State. That additional period shall be a maximum of 6 months and shall cease at the moment when the additional information is received by the Member State. Where at the end of that period the applicant has not submitted the missing elements, the Member State shall inform the applicant that the application is inadmissible.’ [10]. The maximum delay is therefore set at 18 months. However, although clearly defined, these steps are not so straightforward in many cases [51].

### 4.4. Extension of Uses Process

The request for an extension is somehow similar, except the need of support from corresponding agricultural sectors at the deposit step. Some extensions were voted after submission, some others were granted with admissibility and voted rapidly after; some later were following the full approval pathway, including admissibility, evaluation, outcome, full vote at PAFF Committee (appearance in Part A (lecture, discussion), C (proposal) and B (effective vote)). This latter process sometimes takes the same amount of time compared to a new approval, which is considered very excessive by the applicants, having an approved substance at the beginning of their request and only asking for one line sometimes in the Good Agricultural Practices (GAP) table.

### 4.5. Regulation Analysis

The EU Pesticides Database [52] was used to detect basic substances and their status (approved, nonapproved, pending, and modifications of Review Reports). Corresponding linked Implementing Regulations [20] attached to each active substance were found using the same method and cross-verified with Implementing Regulation (EU) 540/2011. The EU law database for Eur-Lex was also used to track each Implementing Regulation publication. Furthermore, EFSA documents were also compiled to extract decisions supportive analyses.

## 5. Conclusions

Searching for alternative products for crop protection is an important strategy for promoting more sustainable food systems. The use of basic substances is in line with the restriction on the application of chemical PPPs and the principles of the European Green Deal and SDGs, mostly renewables and with no MRL. There is relatively poor information about the effectiveness of basic substances as compared to synthetic pesticides and biological PPPs. A higher testing and validation of the use of basic substances as a phytosanitary measure can lead to further reduction of application of synthetic pesticides. In addition, searching for the most effective dosage of the basic substance is critical and an important question for phytosanitary consultants (the plant doctors that are opinion leaders in application of innovations in pest management), growers, stakeholder, and companies to avoid that their application at the recommended dose can lead to a lack of or poor effectiveness of these substances. For this reason, a flexibility might be required in the suggested dosage of basic substances approved to ensure good maintenance of the quality and quantity of production, which is one of the keys of the Farm to Fork Strategy of the European Green Deal. Moreover, a defined timeline for approval is basilar to have the chance to increase the number of basic substances available for growers, the scientific community, and the whole agricultural sector, with final benefits for the consumers.

## 6. Patents

All Implementing Regulations may be considered as patents but with free exploitation, since no Marketing Authorizations are needed for basic substances.

## Figures and Tables

**Table 1:** Application of the basic substances approved.

Basic Substance	Approval Regulation and Applicant	Crops and/or Situation	Function in Plant Protection	Pests or Group of Pests Target	Application				Application Rates				Notes
					Method	Growth Stage & Season	No. Min/Max	IBA ^1^ (Days)	Min–Max	Water L/ha Min–Max	Total Rate	PHI ^1^	
***Equisetum arvense* L.**	Reg. (EU) No 462/ 2014 **ITAB**	Fruit trees Apple fruit (*Malus pumila*, *Malus domestica*) Peach-tree (*Prunus persica*)	Fungicide	Foliar fungi like scab disease (*Venturia inaequalis*), Powdery mildews (*Podosphaera* *leucotricha*) Peach leaf curl (*Taphrina deformans*)	Foliar application spraying	From green leaf tip (BBCH 53) to flowers fading (BBCH 67) Spring	2–6	7	200 g/hL	500–1000	1000–2000 g/ha	Na ^1^	Plant homogenate extracted with hot water and filtered to be used 24 h after preparation
		Grapevine (*Vitis vinifera*)		Downy mildew (*Plasmopara viticola*), Powdery mildew (*Erysiphe necator*)		From 1st shoots (BBCH 10) to cluster tightening (BBCH 57) Spring to summer				100–300	200–600 g/ha	Na	
		Cucumber (*Cucumis sativus*) roots		Powdery mildew (*Podosphaera fusca*) Root fungi like common root rot, seedling blight (*Pythium* spp.)	Root feeding application and foliar application spraying	From (9th leaf unfolded on main stem—BBCH 19) to 9 or more primary side shoots visible (BBCH 49)	2	3–4		300	600 g/ha	15	
		Tomato (*Lycopersicum esculentum*)		Early blight (*Alternaria solani*), Septoria blight (*Septoria lycopsersici*)	Foliar application spraying	First inflorescence visible (BBCH 51) to BBCH 59 summer		14					
		Strawberry (*Fragaria* × *Ananassa*) Raspberry (*Rubus idaeus*)		Gray mold (*Botrytis cinerea*), Powdery mildew (*Podosphaera aphanis*), red core (*Phytophthora fragariae*), other fungi like *Colletotrichum acutatum*	Foliar application spraying ^2^	Growth restart till end of fructification. Early spring till end of summer Stage BBCH 1 to BBCH 89	4–8	5–14	225 g/hL	300	675 g/ha	Na	
		Potato (*Solanum tuberosum*)		Late blight (*Phytophthora infestans*), early blight (*Alternaria solani*), powdery mildew (*Erysiphe cichoracearum*)		Stage BBCH 1 until BBCH 9							
		Ornamental trees use of which *Prunus* spp. Roses *Rosa* spp.		Ornamental fungal diseases, rose black spot (*Marsonia* spp.), Rose rust (*Phragmidium* *mucronatum*), leaf curl diseases, monilioses, oidium and mildew	Included in mulch	Not relevant	1	Na	Na	Na	9000 g/ha		Dry plant aerial parts usage never applied on whole hectare
**Chitosan hydrochloride**	Reg. (EU) 2021/1446 **ChiPro**	Fruits berries and small fruit	Elicitor, having a fungicide and bactericide effect via the stimulation of natural defence mechanisms	Plant elicitor, plant resistance against pathogenic fungi and bacteria	Low–Medium volume spraying	From 1 leaf development (main shoot) to 7 development of fruit	4–8	14	50–200 g/hL	200–400	100–800 g/ha	0	
		Vegetables							50–100 g/hL		100–400 g/ha		
		Cereals											
		Spices											
		Crops for animal feed											
		Cereals Seed treatment			Low volume spraying	Before sowing	1	Na		Na	Na		
		Potatoes Seed treatment			Low volume spraying/dipping					Na	Na		
		Sugar beet Seed treatment							50–200 g/hL	Na	Na		
		Ornamental bulbous plants			Bulb treatment– Dipping/drenching	Germination (BBCH 00–01)			50–100 g/hL	200–800	100–800 g/ha		
					Low–Medium volume spraying	Leaf development– senescence (BBCH 10–92)	1–8	5–7	50–200 g/hL	200–400			
					Low–Medium volume spraying	Leaf development –senescence (BBCH 10–92)							
		Beet crops											
**Sucrose**	Reg. (EU) No 916/2014 **ITAB** **IRBI**	Apple trees/ orchards (*Malus pumila,* *Malus* *domestica*)	Elicitor, having an insecticidal and fungicidal effect via the stimulation of natural defence mechanisms	Fruits borer like Codling moth (*Cydia pomonella*) ^3^	Foliar application spraying early in the morning before 9 AM (Solar time)	From spring BBCH stage 6 to summer BBCH stage 89	7–10	15	10 g/hL	600–1000	60–100 g/ha	Na	Cold water solution prepared just before application
		Sweet Maize (Sweet corn) (*Zea mays* L. convar. *saccharata* Koern)		Corn borer (*Ostrinia nubilalis* Hbn.) ^3^		From the BBCH stage 12 to 89	3–4			200	20 g/ha		
		Maize (corn grain) (*Zea mays* subsp. *mays* (L.)) and corn seed		Corn borer (*Ostrinia nubilalis* Hbn.) ^3^		From the BBCH stage 12 to 51	3–4						
		Grapevine (*Vitis vinifera*)		Vine leafhopper (*Scaphoideus* *titanus*) ^3^		From the BBCH stage 17 to 57	3	7		150	15 g/ha		
		Grapevine (*Vitis vinifera*)		Downy mildew (*Plasmopara viticola*) ^3^		From 1st shoots to cluster tightening spring (BBCH 10–57)	up to 2			100–200	10–20 g/ha		
**Calcium hydroxide**	Reg. (EU) 2015/762 **IFOAM**	Pome fruit	Fungicide	*Neonectria* *galligena*	Sprinkler application	Leaf drops end of October till end of December	2–7	5–14	104–208 L/ha ^4^ 1460 L/ha ^5^	5000–10,000 L/ha	25–50 kg/ha 350 kg/ha ^3^	Na	
		Pome fruit and stone fruit		*Neonectria* *galligena* and other diseases	Spray application				With products at 24% 63–104 L/ha ^4^ 728 L/ha ^5^ with products at 33.12% 45–76 L/ha ^4^ 532 L/h^5^	500–1000 L/ha	15–25 kg/ha ^4^ 175 kg/ha ^5^		
					Brush application directly on pruning wounds and old cancers on stems ^6^	Winter to March	1–2	21	With products at 24% 450 L/ha ^3^ 900 L/ha ^4^ with products at 33.12% 450 L/ha ^4^ 900 L/ha ^5^	No extra water ^6^	149.04 kg ^4^ 299.08 kg ^5^		
**Vinegar**	Reg. (EU) No 540/2011 Reg. (EU) 2015/1108 Reg. (EU) 2019/149 **ITAB**	Wheat seeds (*Triticum vulgare*), common wheat (*Triticum aestivum*), durum wheat (*Triticum durum*), spelt (*Triticum spelta*)	Fungicide, bactericide and herbicide	Common bunt (*Tilletia caries,* *Tilletia foetida*)	Seed treatment just before seeding	Autumn	1	Na	25–50 ^7^ per 100 kg of seed	Not applicable	24–100 ^7,8^	Na	
		Barley seeds (*Hordeum vulgare*)		Barley leaf stripe (*Pyrenophora* *graminea*)									
		Market vegetables Gardening like carrot (*Daucus carota*), tomato (*Solanum lycopersicum*), bell pepper (*Capsicum* spp.)		*Alternaria* spp.		Autumn to spring			Seeds are tem-porary soaked in the dilution then removed		Seeds are temporary soaked in the preparation then removed		
		Market vegetables gardening like tomato (*Solanum* *Lycopersicum*), bell pepper (*Capsicum* spp.), cabbage (*Brassica oleracea*)		*Clavibacter* *michiganensis,* *Clavibacter* *michiganensis* subsp. *michiganensis*, *Pseudomonas* *syringae* pv. *tomato*, *Xanthomonas campestris* pv. *vesicatoria*, *Botrytis aclada*			1			Na			
		White and red chestnut (*Aesculus* L.), *Sycamore* spp. (option), *Acer* spp.		Bacteria: *Pseudomonas* *syringae* pv. *aesculi*	Tools application before sawing or cutting ^9^	Na	1 per day to each time before use	1	400 g/hL	Na	Na		Waiting period 30 s after washing
		Hawthorns (Rosaceae): *Crataegus* spp., *Amelanchir,* *Aronia*, *Chaenomeles*, *Cotoneaster*, *Cydonia*, *Malus, Photinia*, *Potentilla*, *Prunus*, *Pyracantha*, *Pyrus*, *Rosa*, *Sorbus* and *Spiraea*		Fire blight (*Erwinia* *amylovora*)		Na			Na	Na	Na		
		Many ornamental plants including *Acer*, *Cotoneaster*, *Euonymus*, *Forsythia*, *Magnolia*, *Philadelphus*, *Populus*, *Prunus*, *Pyrus*, *Rosa*, *Rubus*, *Syringa* and *Vaccinium*		Bacterial blight /canker (*Pseudomonas syringae* pv. *syringae*)		Na			Na	Na	Na		
		*Plane* sp., *Platanus*, *Prunus* sp., *Chestnut* sp., *Aesculus* L., *Sophora* spp., *Linden* sp., *Tilia*		Rot fungi, especially phellins: *Phellinus*, Tinder polypore and ruffled (*Fomes* *fomentarius*)		Na			Na	Na	Na		
		Elm (elm other than Lutèce) (*Ulmus* spp.)		Vascular fungi: *Ophiostoma* spp.		Na	Na		Na	Na	Na		
		*Maple* sp., *Acer* sp.		Wilt disease		Na	Na		Na	Na	Na		
		*Ailanthe* sp., *Ailanthus* *altissima*		*Verticillium* spp.	Na	Na	Na	Na	Na	Na	Na		
		*Maple* sp., *Acer* sp.; *Sycamore*, *Acer* spp.; *Chestnut* sp., *Aesculus* L.; *Beech* sp., *Fagus* spp.		Sooty-Bark disease (*Cryptostroma* *corticale*)	Na	Na	Na	Na	Na	Na	Na		
	ITAB/ITEIPMAI	Medicinal aromatic and perfume crops		Weeds	Spray ^10^	Pre crop emergence	1	Na	10 kg/hL	100 L vinegar (no dilution)	10 kg/ha	>120	Phytotoxic to plant, may kill the young plants ^11^
	Charbonneaux-Brabant	paths, borders, sidewalks and terraces		Weeds	Direct spray (spot application)	Vegetation Period of the weeds	1–2	7–21	6 kg/hL	100 L (diluted vinegar)	6–12 kg/ha	Na	Temp > 20 °C phytotoxic to plant, may kill the young plants ^12^
***Salix* spp. cortex**	Reg. (EU) 2015/1107 **ITAB**	Fruit trees, Peach tree (*Prunus persica*)	Fungicide	Foliar fungi like *Taphrina deformans*	Foliar application spraying	From 1st shoots (BBCH 10) to cluster tightening (BBCH 57) spring	2–6	7	222.2 g/hL	500–1000 L/ha	1111.1–2222.2 g/ha	Na	Plant homogenate extracted with hot water (infusion), filtered and diluted by 3, to be used up to a maximum of 24 h after preparation. The product cannot be applied in case of hot temperature. It is used in case of rainy period
		Apple fruit (*Malus pumila*, *Malus* *domestica*)		Foliar fungi like scab disease (*Venturia inaequalis*), powdery mildew (*Podosphaera leucotricha*)		From green leaf tip (BBCH 53) to flowers fading (BBCH 67) spring							
		Grapevine (*Vitis vinifera*)		Downy mildew (*Plasmopara viticola*), Powdery mildew (*Erysiphe necator*)		From 1st shoots (BBCH 10) to cluster tightening (BBCH 57) spring to summer				100–300	222.2–666.6 g/ha		
**Lecithins**	Reg. (EU) No 540/2011 Reg. (EU) 2015/1116 **ITAB** **DAE**	Fruit trees Apple fruit (*Malus pumila*) Peach tree (*Prunus persica*)	Fungicide	Powdery mildew (*Podosphaera leucotricha*) Peach leaf curl (*Taphrina deformans*)	Spray application	BBCH 03 to BBCH 79	3–12	5	75 g/hL	500–1000	375–750 g/ha	5	
		Gooseberry Ribes uva-crispa		Powdery mildew (*Microsphaera grossulariae*)		BBCH 10 to BBCH 85	2–4		200 g/hL		1000– 2000 g/ha		
		Market vegetables gardening like cucumber (*Cucumis sativus*)		Powdery mildew (*Podosphaera fusca*)		BBCH 10 to BBCH 89	2–6		150 g/hL	1000– 1500	1500– 2250 g/ha		
		Lettuce (*Lactuca sativa*)		*Erysiphe cichoracearum*			2	7					
		Mash(*Valerianella locusta*)		*Erysiphe polyphaga*			1	Na					
		Tomato (*Lycopersicum* *esculentum*)		Tomato late blight (*Phytophthora infestans*)			2 to 6	7					
		Endive (*Cichorium endivia* L.)		*Alternaria cichorii*									
		Ornamentals, especially roses		Powdery mildew and other fungal diseases			3–12	5	75 g/hL	100–300	75–225 g/ha		
		Grapevine (*Vitis vinifera*)		Downy mildew (*Plasmopara viticola*), Powdery mildew (*Erysiphe necator*)		BBCH 11 to BBCH 85						30	
		Strawberry (*Fragaria* × *Ananassa*) Raspberry (*Rubus idaeus*)		Powdery mildew and other fungal diseases, i.e., *Podosphaera aphanis*, Red core (*Phytophthora fragariae*)		Growth restart till end of fructification Early spring till end of Summer Stage BBCH 10 to BBCH 89 (2nd crop, other strawberries have reached them specific color)			200 g/hL	300–500	600–1000 g/ha	Na	
		Potato (*Solanum tuberosum*)		Late blight (*Phytophthora infestans*)		Stage BBCH 10 until BBCH 90	3–12			100–400	200–800 g/ha		
		Carrot (*Daucus carota* subsp. *sativus*)		Powdery mildew (*Leveillula taurica*)		BBCH 19 to BBCH 90	4	14		1000	2000 g/ha		
**Fructose**	Reg. (EU) 2015/1392 **ITAB** **IRBI**	Apple fruit (*Malus pumila, Malus domestica*)	Elicitor, having an insecticidal and fungicidal effect via the stimulation of natural defence mechanisms	Fruits borer like Codling Moth (*Cydia* *pomonella*) ^13^	Foliar application spraying early in the morning before 9 AM (solar time)	From spring BBCH stage 6 to summer BBCH stage 65	5–7	21	10 g/hL	600–1000	60–100 g/ha	Na	Cold water solution prepared just before application
		Maize (Corn grain) (*Zea* *mays* subsp. *mays* L.) Sweet Maize (Sweet corn) (*Zea mays* L. convar. *saccharata* Koern)		Symphylans (*Scutigerella* *immaculata*) ^13^	Treatment in seedling line before 9 AM (solar time)	-	1	Na		40	40 g/ha		
		*Zea mays* subsp. *mays* L.			Foliar application Spraying early in the morning before 9 AM (solar time)	1 application at 2–3 leaves (BBCH 12–13) + 1 application at 4 leaves (BBCH 14)	2	1–2		82	8.2 g/ha		
		Grapevine (*Vitis vinifera*)		Vine leafhopper (*Scaphoideus* *titanus*) ^4^	Foliar application spraying early in the morning before 9 AM (solar time)	From the BBCH stage 17 to 57	3	3		150	15 g/ha		
		Grapevine (*Vitis vinifera*)		Downy mildew (*Plasmopara viticola*) ^4^		From 1st shoots to cluster tightening Spring (BBCH 10–57)	up to 12	>12		100–200	10–20 g/ha		
**Sodium hydrogen carbonate**	Reg. (EU) 2015/2069 Reg. (EU) 2015/2069 **Danish Environmental** **Protection Agency**	Vegetables Soft fruit Ornamentals	Fungicide and herbicide	Mildews (*Sphaerotheca* spp., *Oidium* spp.)	Broad cast using field spray or greenhouse spray	BBCH 12 to 89	1–8	10	333–1000 g/hL	300–600	2000–5000 g/ha or 0.33–1.0% Max 1% Dose adjusted depending on water volume	1	Different crops have different sensitivity. Check concentrations for phytotoxic effects before widely used
		Grapevine (*Vitis vinifera*)		Powdery mildew (*Erysiphe necator*)	Broadcast using air blast orchard sprayer	BBCH 12 to 89	1–8		420–2000 g/hL	200–600	2500–5000 g/ha or 0.42–2.0%		Volumes and doses will vary according to crop canopy size. Conc. higher than 1–2% can be phytotoxic
		Apple		Apple scab (*Venturia* *inaequalis*)	Broadcast using air blast orchard sprayer	BBCH 10 to 85	1–8		500–1000 g/hL	500–1000	2500–5000 g/ha or 0.5–1.0%		
		Fruit of different types (oranges, cherries, apples, papaya)		Storage diseases like Blue mold (*Penicillium* *italicum*) Green mold (*Penicillium* *digitatum*)	Dipping or surface treatment	Harvested fruit	1–2		1000–4000 g in 100 L water		1–4%		Dose rates between 1–4% has been tested
		Potted plants		Liverwort/ Bryophyte (thallose, *Lunularia* *cruciata*) Green thallus of liverwort plus, fruiting bodies	Direct application of powder	Post emergence late summer or winter	1	Na	Na	Na	122 kg/ha	Na	The product is used for post emergence application. Phytotoxicity of this use was not tested, check on small number of plants before it is widely used
**Whey**	Reg. (EU) 2016/560 **ITAB**	Cucumber (*Cucumis* *sativus*), zucchini squash (*Cucurbita* *pepo*)	Fungicide and virucide	*Podosphaera* *fusca,* *Podosphaera* *xanthii,* *Golovinomyces* *cichoracearum,* *Erysiphe orontii,* *Sphaerotheca* *fuliginea,* *Leveillula* *cucurbitacearum*	Foliar spray ^12^	From three weeks after sowing (9th leaf unfolded on main stem) to 9 or more primary side shoots visible (BBCH 19–49) ^14^	3–5	7	0.6–3 L (0.036 –0.24 kg/hL)	1000–1500	6–30 L (0.36– 2.4 kg/ha)	Na	Whey should be used rapidly after collection, not stored in metal vessel
		Grapevine (*Vitis vinifera*)		Powdery mildew(*Erysiphe necator*)		From 1st shoots to cluster tightening Spring ^15^		7–10	6–30 L (0.36–2.4 kg/hL)	100–30,0 ^15^	6–30 L (0.36– 2.4 kg/ha)		
		Vegetable Gardening, Tomato (*Lycopersicum* *esculentum*)		Tomato (Sinaloa) yellow leaf curl virus Begomovirus		First inflorescence visible Summer (BBCH 10–51) ^15^		3–4	0.6–3 L (0.036 –0.24 kg/hL)	1000– 1500			
		Glove fingertips and mechanical cutting tools All crops		Viruses (Mechanically transferable) e.g., Tobacco mosaic virus (TMV), Tomato mosaic virus (ToMV), Pepper mild mottle virus (PMMV), Cucumber green mottle mosaic virus (CGMMV), Tomato brown rugose fruit virus (ToBRFV)	Dipping	On tools and glove fingertips	Before/after every plant contact ^16^	Na	Na	Na	Na		Dipping for 5 s for gloves and 5 min for mechanical cutting tools. For reasons of efficacy use whey protein powder with at least 80% protein content. Replace the whey solution regularly (e.g., after each crop row) to prevent cross contamination of the plant
**Diammonium phosphate**	Reg. (EU) 2016/548 **ITAB**	Orchards including cherry tree (*Prunus* spp.)	Attractant	Mediterranean fruit fly (*Ceratitis capitata*), Cherry fly (*Rhagoletis cerasi*)	Placed in physical traps	Na	Mass trapping: 1 trap per tree up to 100 traps/ha	42–56 ^17^	max 4 kg/hL	Mass trapping: max 100	Mass trapping: max 4 kg/ha	Na	
		Olive trees (*Olea europaea*)		Olive fly (*Bactrocera oleae*)									
		*Citrus* spp.		Mediterranean fruit fly (*Ceratitis capitata*)									
		Other crops where *C. capitata* cause damage											
**Sunflower oil**	Reg. (EU) 2016/1978 **ITAB**	Tomato (*Lycopersicum* *esculentum*)	Fungicide	Tomato powdery mildew (*Pseudoidium neolycopersici*)	Foliar application spraying	BBCH 32–37 then BBCH 61–71	2 to 4	8	0.092 kg/hL (0.1 L) –0.46 kg/hL (0.5 L)	500 to 1000	0.46 kg/hL (0.5 L)– 4.6 kg/hL (5 L)	2	Precautions must be taken to avoid overwatering and spilling of the dispersion. Treatment should be avoided during flowering time
***Urtica* spp.**	Reg. (EU) 2017/419 **ITAB**	Fruit trees Apple tree (*Malus domestica*), Plum tree (*Prunus domestica*), Peach tree (*Prunus persica*), Red currant (*Ribes rubrum*), Walnut tree (*Juglans* sp.), Cherry tree (*Prunus* sp.)	Insecticide, fungicide, acaricide	Peach-potato Aphid (*Myzus persicae*, *Macrosiphum* *rosae*), wolly Apple aphid (*Eriosoma* *lanigerum*), Currant aphid (*Cryptomyzus ribis*), Walnut aphid (*Callaphis juglandis*), Black cherry aphid (*Myzus cerasi*)	Foliar spraying or Shoot spraying Directly on aphids	Spring summer until BBCH 87 (fruit ripe for picking)	1–5	7–15	1500 g/hL (dry matter) ^18^	300–900 L/ha	4500–13,500 g/ha ^17^	7	Preventive treatment is inefficient 24 h of maceration at 20 °C is enough
		Bean, for example French bean (*Phaseolus vulgaris*)		Black bean aphid (*Aphis fabae*)		Spring Summer until BBCH 89 (fully ripe)				300–500 L/ha ^18^	4500–7500 g/ha ^18^		
		Potato (*Solanum tuberosum*)		Peach-potato aphid (*Myzus persicae*)	Na	Spring Summer until BBCH 49 (end of tuber formation)	Na	Na	Na		4500–10,000 g/ha ^17^	Na	
		Leaf Vegetables: Lettuce (*Lactuca* *sativa*), Cabbage (*Brassica olaeracea*)		Aphids, for example: cabbage aphid (*Brevicoryne brassicae*), *Nazonoviaribis nigri*)	Foliar spraying or shoot spraying directly on aphids	Spring Summer until BBCH 19 (9 or more true leaves unfolded)	1–5	7–15	1500 g/hL (dry matter) ^18^		4500– 7500 g/ha ^18^	7	Preventive treatment is inefficient 24 h of maceration at 20 °C is enough
		Elder tree (*Sambucus racemosa*)		Elder aphid (*Aphis sambuci*)		Spring Summer				400–800	6000– 12,000 g/ha ^18^		
		Rose (*Rosa* sp.)		Rose aphid (*Macrosyphum rosae*)						300–600	4500–9000 g/ha ^18^		
		*Spiraea* sp.		*Aphis spiraephaga*									
		Brassicaceae (cabbage—*Brassica oleracea*, rapeseed— *Brassica napus*, radish— *Raphanus sativus*)		Fleabeetle (*Phyllotreta nemorum*)	Foliar spraying	Spring Summer Until BBCH 19 (9 or more true leaves unfolded	1–6			300–500	4500–10,000 g/ha ^18^		
				Diamondback moth (*Plutella xylostella*)		Spring Summer until BBCH 49 (Typical leaf mass reached)							
		Apple tree (*Malus domestica*), Peer tree (*Pyrus communis*)		Codling moth (*Cydia pomonella*)		2 treatments in April, 1 treatment in May	3	15		300–900	4500–13,500 g/ha ^18^		
		Bean, for example French bean (*Phaseolus vulgaris*)		Two-spotted spider mite (*Tetranychus urticae*)		Spring Summer Until BBCH 89 (fully ripe)	1–6 (commonly 3)	7–21		300 –500	4500–7500 g/ha ^18^	7	24 h of maceration at 20 °C is enough
		Grapevine (*Vitis vinifera*)		Two-spotted spider mite (*Tetranychus urticae*), red spider mite (*Tetranychus telarius*)		Spring Summer Until BBCH 89 stage	1–6 (three before flowering, three after flowering)			300–600	4500–9000 g/ha ^18^		
		Brassicaceae (Mustard family, *Brassica* sp., *Sinapis* sp., radish— *Raphanus sativus*)		*Alternaria* sp.	Foliar spraying	Spring Summer until BBCH 49 (typical leaf mass reached)	1–6	7–15	1500 g/hL (Based on dry matter) ^18^	300–500	4500–7500 g/ha ^18^	7	
		Cucurbitaceae (Cucumber— *Cucumis sativus*)		Powdery mildew (*Erysiphe polygoni*)*,* *Alternaria alternata* f. sp. *cucurbitae*		Until BBCH 89 (typical fully ripe colour)							
		Fruit trees (Apple trees— *Malus domestica*, Plum trees— *Prunus domestica*, Peach trees– *Prunus persica*, Sweet cherry tree— *Prunus avium*)		Leaf spot (*Alternaria alternata*), brown rot, blossom blight (*Monilinia laxa*), *Botrytis cinerea*, back bread mold (*Rhizopus stolonifer*)	Foliar and Fruit spraying	Spring Summer Until BBCH 87 (fruit ripe for picking)				300–900	4500–13,500 g/ha ^18^		
		Grapevine (*Vitis vinifera*)		Downy mildew (*Plasmopara viticola*)	Foliar spraying	Spring Summer Until BBCH 89 stage			1500 g/hL (Dry matter) ^19^	300–600	4500– 9000 g/ha ^18^		
		Potato (*Solanum tuberosum*)		Late blight (*Phytophthora infestans*)		Spring Summer Until BBCH 49 (End of tuber formation)				300–500	4500–7500 g/ha ^18^		
		Cucumber roots (*Cucumis* *sativus*)		Powdery mildew (*Podosphaera fusca*), Root fungi like common root rot, seedling blight (*Pythium* spp.)	Included in mulch	Not relevant	1	Na	Na	Na	15 kg/ha ^18^	Na	Dry plant aerial parts
		Tomato (*Lycopersicum* *esculentum*)		Early blight (*Alternaria solani*), Septoria blight (*Septoria lycopsersici*)									
		Ornamental trees use of which *Prunus* spp. Roses (*Rosa* spp.)		Ornamental cryptogramic diseases Rose black spot (*Marsonia* spp.), Rose rust (*Phragmidium mucronatum*), leaf curl diseases, monilioses, Oidium and mildew									
**Clayed charcoal**	Reg. (EU) 2017/428 **Ets Christian Callegari**	Grapevine (*Vitis vinifera*)	Protectant	Esca (black measles) caused by a complex of fungi that includes several species of *Phaeoacremonium* primarily by *Phaeoacremonium minimum* (Pm) (currently known as *P. ultimum*), and by *Phaeomoniella* *chlamydospora* (Pch)	Soil burying	Na	1/3 years	1095	Na	Na	500	Na	
**Hydrogen peroxide**	Reg. (EU) 2017/409 **ITAB**	Vegetables—*Solanaceae* like tomato (*Lycopersicon esculentum*), bell pepper (*Capsicum* spp.)	Fungicide, bactericide	Soil bacteria (*Ralstonia solanacerum*), *Botrytis cinerea*	Apply before cutting	Na	To be applied before every use of the tool	Na	Na	Na	Na	Na	Waiting period 30 s after washing
		Lettuce (*Lactuca sativa*)		Bacterial leaf spot pathogen (*Xanthomonas* *campestris* pv. *vitians*)	Seed treatment before sowing ^19^	Na	1						Seeds are immersed in the prepared solution for 5 to 15 min (seed treatment)
		Horticulture flowers like common zinnia (*Zinnia* *elegans*)		Fungi, especially pathogenic *Alternaria* *zinnia*, *Alternaria* *alternata,* *Fusarium* spp.									
**Sodium chloride**	Reg. (EU) 2017/1529 Reg. (EU) 2021/556 **ITAB** **AHDB**	Grapevine (*Vitis vinifera*)	Fungicide, insecticide, herbicide	Fungal diseases Powdery mildews (*Erysiphe* *necator*)	Foliar application spraying	From 1st shoots (BBCH 10) to cluster tightening (BBCH 57) Spring to summer	1–2	Na	600– 2000 g/hL	200	1200–4000	30	In case of 2 applications: one at 20 g/L + one at only 10 g/L. Maximum total rate of salt shall not exceed 6 kg/ha per year. Careful application should be controlled in terms of spray and target should be only the foliage. Low volumes are recommended in order to avoid spill. It is recommended not to spray every year, only in emergency cases. Maximum total rate of sodium chloride shall not exceed 6 kg/ha per year
		Mushrooms like *Agaricus* *bisporus*		Fungal diseases like cobweb disease (*Cladobotryum* strains—i.e., *Mycophilum*), dry bubble disease (*Lecanicillium* *fungicola*), wet bubble disease (*Mycogone* *perniciosa*)	Hand trowel cup scoop	On finding the pathogen. No earlier than 16 days into grow cycle	1	Na	0.03 g/kg	–Dry	80–100 g/ha	Na	Salt is used as a spot treatment to cover incidents of disease. On a well-managed farm, disease will be spotted early with specialist teams identifying and spot treating. This avoids harvesters accidently spreading disease thorough contamination of personal protective equipment (PPE) and transfer to other areas. This in turn will keep on site disease levels low and avoid the use of large volumes of salt.
		Grapevine (*Vitis vinifera*)		European grapevine moth (*Lobesia* *botrana*)	Foliar application spraying	1st late April to May (BBCH 55–57) 2nd July (BBCH 75–77) 3rd September (BBCH 83–91)	1–3	Depen-ding on egg stage	600 g/ha	200	1200–3600 g/ha	30	Careful application should be controlled in terms of spray and target should be only the foliage. Low volumes are recommended to avoid spill. It is recommended not to spray every year, only in emergency cases
		Salt swamps and salt marshes		*Baccharis* *halimfolia*	Spot application on drilled tree stump or on soil in direct vicinity of tree stump	November –February	1	Na	Na	Na	10–100 g per tree stump ^20^	Na	Treatment is allowed only in salt marshes and salt swamps zones as defined by national or local authorities. Treatment should be performed outside the rainy period
**Beer**	Reg. (EU) 2017/2090 **ITAB**	All edible and nonedible crops	Molluscicide	Pest slugs and snails	Specific traps for slugs	At the beginning of infestation	1–5	Na	Not applicable (because ready to use liquid)	Na	Na	Na	
**Mustard seed powder**	Reg. (EU) 2017/2066 **ITAB**	Wheat seeds (*Triticum* *vulgare,* *Triticum aestivum*), Durum wheat (*Triticum* *durum*), Spelt (*Triticum* *spelta*)	Fungicide for seed treatment	Fungi like Common Bunt (*Tilletia caries,* *Tilletia foetida*)	Seed application before sowing	Summer to Autumn	1	Na	Na	Na	1.5 kg/100 kg seeds	Na	Mix 1.5 kg of mustard seeds powder with 4.5 L water. Treat 100 kg seeds with the slurry created
**Talc E553B**	Reg. (EU) 2018/691 **COMPO Expert France SAS**	Fruit trees i.e., Apple fruit (*Malus* *Domestica*), Pear tree (*Pyrus* sp.), Olive tree (*Olea* *europea*), etc.	Insectifuge, fungifuge	Physical barrier, Insectifuge: Insects and mites like *Cacopsylla pyri*, *Cacopsylla* *fulguralis*, *Drosophila* *suzukii*, *Panonychus* *ulmi*, *Bactrocera oleae*	Foliar application spraying	From BBCH 41	2–5	21–28	1st application: 2.13 to 3.54 kg/hL succeeding applications: 1.7 to 2.83 kg/hL	600–1000	1st application: 21.25 kg/ha succeeding applications: 17 kg/ha	Na	Water solution prepared just before application and maintained stirred
		Fruit trees i.e., Apple fruit (*Malus* *Domestica*), Pear tree (*Pyrus* sp.)		Physical barrier, Fungifuge: Foliar fungi like mildews (*Venturia inaequalis*, *Erysiphe necator*)			3–5	14–21	1.28–2.13 kg/hL		12.75 kg/ha		
		Grapevine (*Vitis vinifera*)				From BBCH 20	2–5	21–28	4.25–8.5 kg/hL	150–300			
**Onion oil**	Reg. (EU) 2018/1295 **Bionext**	Carrots, celery, parsnip, parsley root	Repellent, scent masking	Carrot root fly (*Psilla rosae*)	Masking the smell of the umbelliferous crop by onion oil evaporated from dispensers	Shortly after planting or crop emergence (around mid–April) until end of November (before harvest)	1	Na	Na	Pot dispensers 0.08–0.160 L/ha Granule Dispenser 17.6–35.2 g/ha	Na	Na	4–8 dispensers per ha professional use only
**L-cysteine**	Reg. (EU) 2020/642 **Soleo-EcoSolutions**	All crops and forestry in tropical areas	Insecticide	Leaf cutting ants	Hand held spreader	Post swarming (July)	1–3	30	3–36 kg granules/ha	Na	Min 0.015 kg/ha Max 2.88 kg/ha ^21^	Na	Used as an insecticide against ants. Application is made by hand on nest of ants. The application can be renewed, if necessary, with a maximum of 3 applications. Minimum/Maximum number of nests by hectare: 10–120
**Cow milk**	Reg. (EU) 2020/1004 **Basic-Eco-Logique**	Grapevine (*Vitis vinifera*)	Fungicide and virucide	Powdery mildew (*Erysiphe necator*)	Foliar application Spraying	From 1st shoots (BBCH 07) to inflorescences fully developed; flowers separating (BBCH 57) ^22^	3–6	6–8	10–40 L/hL	100–300	10–120 L/ha	Na	
		Vegetable Gardening pumpkin (*Cucurbita pepo*)		Pumpkins powdery mildew (*Podosphaera fusca*)		From leaf development (BBCH 01) until flowering (BBCH 06) ^23^	3–4	7–12	50 L/hL	400	200 L/ha		No application in presence of fruits
		Flower Gerbera (*Gerbera* *jamesonii*)		Powdery mildew (*Erysiphe* *cichoracearum*)		Before and during flowering (BBCH 51–69)	3–4	7	16 L/hL	500–1000	80–160 L/ha	8	
		Cucumber (*Cucumis sativus*), Zucchini squash (*Cucurbita pepo*)		Powdery Mildew (*Podosphaera fuliginea*)		From three weeks after sowing (9th leaf unfolded on main stem) to 9 or more primary side shoots visible (BBCH 19–49) ^24^	3–4		5–10 L/hL	1000–1500	50–150 L/ha	Na	
		Soybean (*Glycine max* (L.) Merr)		Soybean Powdery mildew (*Erysiphe diffusa*)		On leaves (BBCH 19–49)	3–4	7	18 L/hL	1000–1500	180–270 L/ha		
		Glove fingertips and mechanical cutting tools All crops		Viruses (mechanically transferable) e.g., Tobacco mosaic virus (TMV), Tomato mosaic virus (ToMV), Pepper mild mottle virus (PMMV), Cucumber green mottle mosaic virus (CGMMV)	Dipping	On tools	Before/after every plant contact	Before/after every plant contact	Before/after every plant contact	Na	Na		Dipping for 2 s. For reasons of efficacy use milk with at least 3,5% protein content. Replace the milk regularly (e.g., after each crop row) to prevent cross-contamination of the plants
***Allium cepa* bulb extract**	Reg. (EU) 2021/81 **ITAB**	Potatoes (*Solanum* *tuberosum*)	Fungicide	Early blight (*Alternaria solani*)	Spray	BBCH 21–85	3–5	7	1 kg/hL	600– 1000	6–10 L/ha (0.3–0.5 kg onion bulb/ha)	Na	
		Vegetable Gardening Tomato (*Lycopersicum esculentum*)		Tomato late blight (*Phytophthora* *infestans*)		75 days after planting BBCH 21–75		3–4		1500	15 L/ha (0.75 kg onion bulb/ha)		
		Cucumber (*Cucumis sativus*)		Cucumber gray mold (*Botrytis cinerea*)				7					
**Chitosan**	Reg. (EU) 2022/456 **KitoZyme**	Horticulture	Fungicide	Plant elicitor, plant resistance against pathogenic fungi and bacteria	Spray Low–Medium volume spraying	BBCH 09 to BBCH 89	4–8	2 weeks	50–100 g/hL	200–400	100–400	Na	Chitosan can be prepared for use following any of the two recipes provided in Appendix of Reg. (EU) 2022/456 (preparation for use).
		olive trees				From 1st new leaf development BBCH 10 to development of fruit BBCH 71					800–3200		
		grapes								200–600	800–7800		
		grass				BBCH 09 to BBCH 89				200–400	800–3200		
		postharvest fruit treatment		Pathogenic fungi and bacteria	Immersion	Postharvest BBCH 89+	1	-	1	-	-		

^1^ IBA: Interval between applications; PHI: minimum preharvest interval; Na: Data not available; ^2^ The product cannot be applied in case of hot temperature. It is used in case of rainy period; ^3^ Indirect actions, no direct insecticide and fungicide properties; ^4^ maximum of rate per application; ^5^ maximum total rate per crop/season; ^6^ The aqueous solutions in this application are applied with few or without dilution. Here the case without dilution is calculated. Usually, not all trees are treated with brush application but only injured trees. In the calculation of maximum rate, it was assumed that 3000 trees per ha are treated with 0.15 L product per tree. This means that all trees of an orchard would be treated with several big wounds, which would be really the maximum rate and in reality, is very improbable; ^7^ Expressed as acetic acid. 1/1 dilution of vinegar/water L/L; ^8^ Considering 0.9 to 2 qt of seeds per ha; ^9^ Expressed as acetic acid. 50 mL/1 L dilution of vinegar/water for vinegar at 8% acetic acid; ^10^ Of main active substance acetic acid for vinegar at 10% acetic acid; ^11^ Expressed as acetic acid in a preparation with 60% vinegar (diluted in water), for vinegar at 10% acetic acid; ^12^ Treatments must be delayed 24–48 h or more after rain; ^13^ Spray when there is sun (preferably morning); ^14^ Do not apply when any plant is at a later growth stage than BBCH 49; ^15^ With a maximum of 10% concentration (30 L in 300 L); ^16^ Do not apply on treating fingertips right before or during harvest of edible commodities; ^17^ Depending upon environmental factors such as climate and topography; ^18^ The quantities of fresh nettle (or dry matter) written represents the quantities of nettle used in the recipe, but not the quantities that are effectively put in field—there is a filtration before; ^19^ Treatment, just before sowing; ^20^ Assuming plant density of between 0.1/m^2^ to 1/m^2^; ^21^ 300 g of granules per nest multiplied by 120 nest/ha = 36 kg product/ha. Considering a maximum of 8% L-cysteine in the product, the maximum application rate per treatment of L-cysteine is 2.88 kg/ha; ^22^ Do not apply when any plant is at a later growth stage than BBCH 57; ^23^ Do not apply when any plant in the greenhouse is at a later growth stage than BBCH 06 and in presence of fruits; ^24^ Do not apply when any plant in the greenhouse is at a later growth stage than BBCH 49.

**Table 2:** Typical uses of the basic substances.

Substance Name	Use(s)	Application	Recipe	Formulation Type
*Equisetum arv-ense*	Fungicide	Spraying on crops	2–2.25% water dilution 200 to 225 g/100 L water The product cannot be applied in case of hot tem-perature. It is used in case of rainy period	Dispersible concen-trate
		Dry	9 kg/100 kg mulch	Mulch
Chitosan hydrochloride	Elicitor	Spraying on crops or seeds	0.05–0.2% water dilution 50 to 200 g/100 L water Must be applied within 24 h	Soluble powder, paste
Sodium hydrogen carbonate	Fungicide	Aerial parts spraying	0.33–2% water dilution 333 to 2000 g/100 L water	Soluble powder
		Postharvest dipping	1–4% water dilution 1 to 4 kg/100 L water	
	Herbicide	Direct dusting	10 g for a 50 cm Ø pot	Dry powder
Sunflower oil	Fungicide	Foliar spraying	0.1–0.5% water dilution 100 to 500 mL/100 L water	Oil dispersion
Hydrogen peroxide	Seed treatment	Seeds soaking	Ready-to-use solution (<5%)	Ready-to-use solution
*Urtica* spp.	Fungicide Insecticide	Spraying	3–4 days maceration in water at 20 °C Fresh leaves (75 g/L) or dried leaves (15 g/L) Water dilution by 6 of filtered maceration	Dispersible concentrate
		Mulch incorporation	Addition of dried aerial parts. 83 g/kg of mulch	Mulch
Clayed charcoal	Protectant	Soil burying	Buried. 500 kg/hectare maximum	Pellet
Sodium chloride	Fungicide Insecticide	Foliar spraying	0.6–2% water dilution 600 to 2000 g/100 L water	Soluble powder
		Substrate burying	Mix salt in the substrate. 30 g/kg substrate (3%)	Pellet
Beer	Molluscicide	Trap	Covered slug traps. 1 trap per m^2^ maximum	Pure product
Di Ammonium Phosphate	Attractant	Trap	Place in traps/bottle, 30 g/L.	Soluble powder
Onion oil	Odor mask	Oil dispenser	Fill the dispenser with onion oil only (20 mL) Fill the dispenser with oil then add the pellets (4.4 g oil per 30 g granule)	Oil or pellet
L-cysteine	Insecticide	Hand-held spreader	Mixture with matrix (flour, food grade) at a concentration of maximum 8%	Bait (ready for use)
Cow milk	Fungicide	Foliar spraying	5–50% water dilution = 0.5 to 5 L of cow milk filled up with water to 10 L	Soluble concentrate
		Dipping	Dipping tools for 2 s in undiluted cow milk. For reasons of efficacy use milk with at least 3.5% protein content	
*Allium cepa* L. bulb extract	Fungicide	Spray application	Boil 500 g of chopped onions in 10 L of water for ten minutes then let infuse for a quarter of an hour and filter the mixture	Dispersible concentrate
Chitosan	Fungicide	Spray application & Immersion	Preparation 1: added to a half-filled water tank, making sure the powder is evenly distributed over the water surface to avoid aggregation. The mixture should be stirred vigorously while adding the remaining water. The mixture should be used as soon as possible. Preparation 2: dissolved in water with pH < 5. The pH of water should be regulated by adding 7 mL vinegar (8% of acetic acid) per 1 L of water).	Soluble powder
Vinegar	Fungicide	Seed treatment	Vinegar to be diluted in compliance with the rates of application reported in Appendix II. Undiluted for uses as herbicide on medicinal aromatic and perfume crops. For the herbicidal use in spot applications on paths, borders, sidewalks and terraces, vinegar needs to be diluted to a concentration of 60% vinegar in water (60/40 vinegar/water).	Liquid for seed treatment
		Tools disinfection		
	Herbicide	Spray or spot application/		Liquid
	pH modifier	In combination with chitosan		Liquid

Some applications were not validated by DGSanté and Member States during discussion and votes. Some were withdrawn (Table 3) by applicants during evaluation or discussions with no regulatory trace, while some were processed up to the vote and finally non-approved with corresponding Implementing Regulations (Table 4).

**Table 3:** Basic substance applications retired during the evaluation process.

Basic Substances Removed/Withdrawn during Evaluation			
Substance Name	Intended Use(s)	EFSA Opinion	Reason(s)
***Castanea*** **and *Schinopsis* sp. tannins**	Bactericide, fungicide and nematicide	EN 1363	Limited number of studies about toxicity and residues led to a doubt concerning exposure assessment. Non-dietary exposure considered as hazardous
**Honey from rhododendron**	Rodenticide	EN 1155	Lack of studies concerning substance composition and efficacy on rodents. Rodents in traps might suffer ‘too long’
**Extract from rhododendron**	Rodenticide	EN 1596	Lack of studies concerning substance composition and efficacy on rodents. Rodents in traps might suffer ‘too long’
***Quassia*** ***amara* extract**	Insecticide and repellent	EN 1382	Data gaps were identified for genotoxicity, residues, environmental risk and exposure assessment. Concerns were raised regarding reproductive and endocrine toxicity
***Valeriana officinalis***	Frost protection	None	Potential neurotoxicity, Valerian herbal tea makes it easier to fall asleep
***Citrus pulp***	-	None	-
***Potassium metabisulfite***	-	None	-
***Didecyl-dimethylammonium chloride (DDAC)***	-	RN-214	Toxic to aquatic organisms

**Table 4:** Basic substance applications refused (non-approval).

Substances Not Approved by the European Commission				
Substance Name	Intended Use(s)	Implementing Regulation	EFSA Opinion	Reason(s)
*Achillea millefolium* L.	Fungicide and insecticide	EU no. 2017/2057	EN 1093	Risk assessment for toxicology and ecotoxicology not comprehensive enough left doubts and substance is not considered as foodstuff
*Arctium lappa* L. aerial parts	Fungicide and insecticide	EU no. 2082/2015	EN 699	Risk assessment for toxicology and ecotoxicology not comprehensive enough left doubts and substance is not considered as foodstuff
*Artemisia absinthium* L.	Fungicide, nematicide and insecticide	EU no. 2015/2046	EN 665	Risk assessment for toxicology and ecotoxicology not comprehensive enough left doubts and Regulation (EC) 1334/2008 fixes limits for this substance
*Artemisia vulgaris* L.	Insecticide/repellent	EU no. 2015/1191	EN 644	Risk assessment for toxicology and ecotoxicology not comprehensive enough left doubts and Regulation (EC) 1334/2008 fixes limits for this substance
*Capsicum annuum* L. var. *annuum*, *longum* group, cayenne, extract (Oleoresin capsicum)	Repellent	EU no.2021/464	EN 1838	Risk assessment for toxicology show genotoxicity, causing serious eye damage, being harmful if swallowed and also as cause of skin irritation, although substance is considered as foodstuff
Caffeine	Molluscicide	EU no. 2022/xx	EN 6423	Proposal for non-approval under discussion
Carbon dioxide	Rodenticide	EU no. 2021/80	None	-
Comfrey steeping	Fungicide and insecticide	EU no. 2021/809	EN 1753	Risk assessment for toxicology and ecotoxicology not comprehensive enough left doubts and Regulation (EC) 1334/2008 fixes limits for this substance
Dimethyl Sulfide	Attractant	EU no. 2021/1451	EN 1911	Risk assessment for toxicology and ecotoxicology not provided for long-term toxicity and carcinogenicity concern
Grape (*Vitis vinifera*) cane tannins	Fungicide	EU no. 2020/29	EN 1414	Risk assessment for toxicology and ecotoxicology not comprehensive enough left doubts and substance is not considered as foodstuff
Landes pine tar	Protectant and repellent	EU no. 2018/1294	EN 1311	It may contain substances of concern, so there is a lack of data, so risk assessment is not comprehensive enough and left doubts
*Origanum vulgare* L. essential oil	Fungicide, bactericide and insecticide	EU no. 2017/241	EN 1054	Risk assessment for toxicology and ecotoxicology not comprehensive enough left doubts
Paprika extract E160c	Repellent	EU no. 2017/2067	EN 1096	Risk assessment for toxicology and ecotoxicology not comprehensive enough left doubts
Potassium sorbate	Fungicide	EU no. 2017/2058	EN 1232	Lack of data concerning residues lead to an impossibility concerning exposition assessment
Propolis (water soluble extract)	Fungicide and bactericide	EU no. 2020/640	EN-1494	Defined as a skin sensitizer, risk assessment for genotoxicity and endocrine disruption toxicity left doubts. No safe limit for the use. Substance is not considered as foodstuff
*Rheum officinale* roots extract	Fungicide	EU no. 2015/707	EN 617	Risk assessment for toxicology and ecotoxicology not comprehensive enough left doubts and substance is not considered as foodstuff
*Saponaria* *officinalis* L. roots	Acaricide and elicitor	EU no. 2020/643	EN 1263	Risk assessment for toxicology and ecotoxicology not comprehensive enough left doubts
*Satureja montana* L. essential oil	Fungicide and bactericide	EU no. 2017/240	EN 1051	Risk assessment for toxicology and ecotoxicology not comprehensive enough left doubts
*Tanacetum vulgare* L.	Repellent	EU no. 2015/2083	EN 666	Risk assessment for toxicology and ecotoxicology not comprehensive enough left doubts and substance is not considered as foodstuff
Willow bark and stem extract	Plant growth and defense elicitor	EU no.2022/	EN 1872	Previously proposed for non-approval since not sold for other uses, proposal under discussion, may be accepted.

**Table 5:** Examples of requests from the retailer of the amount of the Maximum Residue Level (MRL) and Acute reference doses (ARfD).

Retailer		Max. %MRL/ Active Substance	Max. Sum %MRL/Sample	Max. %ARfD/Active Substance	Max. Sum %ARfD/Sample	Max. Number of Active Substances /Samples
ALDI/ HOFER	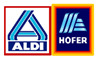	70%	80%	70%	80%	3–5
**ALBERT HEIJN**	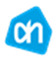	50%	-	50%	-	-
**ASDA**	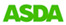	80%	-	-	-	-
BILLA	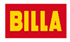	100%	-	100%	-	-
DOHLA	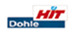	-	70%	-	70%	3–5
EDEKA	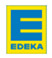	70%	-	100%	-	5
EDEKA OWN BRANDS		50%	-	70%	-	5
GLOBUS	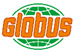	70%	-	70%	100%	5
LIDL	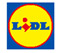	33.3%	80%	100%	-	5
KAUFLAND	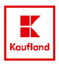	33.3%	80%	50%	50%	5
NORMA	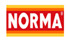	-	70%	-	70%	5
METRO	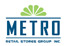	50%	80%	70%	100%	5
**MIGROS**	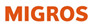	-	-	-	-	6
NETTO	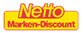	70%	-	100%	-	5
REWE	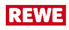	50%	100%	70%	100%	5
REWE OWN BRANDS		50%	100%	50%	-	5
TEGUT	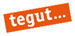	70%	-	70%	-	Max. 4 (>0.01 mg/kg)
TENGEL MANN	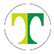	70%	150%	70%	100%	-

**Table 6:** Examples of the applications of the basic substances in research projects.

Substance Name	Use(s)	Program	Reference
**Horsetail (*Equisetum arvense* L.)**	Fungicide	Casdar ‘4P’ Coppereplace	[24,25,26]
**White willow bark (*Salix cortex*)**	Fungicide	Casdar ‘4P’	[24,25]
**Vinegar**	Seed treatment	Casdar ‘Carie’	[27]; http://itab.asso.fr/programmes/carie-ble.php, accessed on 23 May 2022.
**Mustard seed powder**	Seed treatment		
**Sucrose**	Elicitor	Ecophyto ‘Usage’ and Casdar ‘Sweet’, ABAPIC	[28]; https://ecophytopic.fr/cuivre-viticulture/proteger/micro-doses-de-sucre, accessed on 23 May 2022. [29]; https://ecophytopic.fr/sites/default/files/USAGE.pdf, accessed on 23 May 2022.
**Fructose**	Elicitor		
**Lecithin**	Fungicide	Casdar ‘HE’	[30]; https://ecophytopic.fr/recherche-innovation/proteger/projet-he, accessed on 23 May 2022.
**Talc**	Fungicide	out of program	[31]
**Whey**	Fungicide		[32]
**Di-ammonium phosphate (DAP)**	Attractant		[33]; https://ecophytopic.fr/pic/proteger/proteger-ses-oliviers-de-la-mouches-en-limitant-les-traitements, accessed on 23 May 2022.
**Calcium hydroxide**	Fungicide		[34]; https://www.researchgate.net/publication/279636728_The_post-infection_activity_of_hydrated_lime_against_conidia_of_Venturia_inaequalis, accessed on 23 May 2022.
**Chitosan hydrochloride**	Fungicide	Vitinnova	[35]; www.vitinnova.it/en, accessed on 23 May 2022.
		Euphresco BasicS	[16]; https://www.researchgate.net/project/EUPHRESCO-Basic-substances-as-an-environmentally-friendly-alternative-to-synthetic-pesticides-for-plant-protection-BasicS, accessed on 23 May 2022.
		PRIMA StopMedWaste	[36]; www.stopmedwaste.eu, accessed on 23 May 2022.
		ZeroSprechi	[37]; www.zerosprechi.info/en/zerosprechi, accessed on 23 May 2022.
		CleanSeed	[38]; https://www.cleanseed.it/en/cleanseed-2/, accessed on 23 May 2022.

Each use of plant extracts and natural products, such as decoctions, herbal teas, or aqueous solutions, have been defined and tested in the field or identified from the literature then controlled or cross-referenced with producer surveys. Whenever water is mentioned in these tests, it is either natural spring water or rainwater. Each basic substance preparation is described in Section 2.5 of Basic substances applications in EU 2012. The evaluation process of the basic substance application is getting longer, and legal delays fixed by EC are not consistently respected. The evaluation process lasts an average of 19 months (Supplementary Table S1 and Figure S1), while the legal maximum delay is fixed at 18 months until basic substance application admissibility. Even not considering admissibility evaluation delays that are considered outside of the evaluation process, this process becomes longer from year to year, resulting in a delay in availability of additional basic substances.

## Data Availability

Data is contained within the article or supplementary material.

## Supplementary Materials

The following supporting information can be downloaded at: https://www.mdpi.com/article/10.3390/molecules27113484/s1, Figure S1: Time needed for Basic Substance Application admissibility evaluation over time (bars) and tendency line (dotted line); Table S1: Total time of basic substance application process within admissibility to Implementing Regulation publication in months.
