# Supplementary material for: Basic Substances, a Sustainable Tool to Complement and Eventually Replace Synthetic Pesticides in the Management of Pre and Postharvest Diseases: Reviewed Instructions for Users
Source: Molecules. 2022 May 28;27(11):3484. doi: 10.3390/molecules27113484 (PMC9182541; doi:10.3390/molecules27113484)
Supplement: Supplementary file 1 [file molecules-27-03484-s001.zip › molecules-1707225-supplementary.pdf]

Supplementary materials

Table S1. Total time of basic substance application process within admissibility to Implementing Regulation publication in months.

Deposit year	Approval year	Basic substances	Number of months: deposit → approval
**2011**	2014	*Equisetum arvense**	22
**2011**		Chitosan hydrochloride*	22
**2013**		Sucrose (saccharose)*	9
**2014**	2015	*Arctium lappa***	16
**2013**		*Artemsia absinthium***	19
**2013**		*Artemisia vulgaris***	23
**2012**		Calcium hydroxide*	26
**2014**		Fructose*	15
**2013**		Lecithins*	16
**2013**		*Rheum off.* root extract**	17
**2013**		*Salix* spp. cortex*	20
**2014**		Sodium hydrogen carbonate*	13
**2013**		*Tanacetum vulgare***	19
**2013**		Vinegar*	20
**2014**	2016	Diammonium phosphate*	12
**2015**		Sunflower oil*	13
**2015**		Whey/Lactoserum*	10
**2015**	2017	*Achillea millefolium***	19
**2016**		Beer*	11
**2015**		Clayed charcoal*	14
**2015**		Hydrogen peroxide*	11
**2016**		Mustard seed powder*	16
**2015**		*Origanum vulgare* HE**	14
**2015**		Paprika extract E160c**	21
**2015**		Potassium sorbate**	8
**2015**		*Satureja montana* HE**	14
**2016**		Sodium chloride*	13
**2015**		*Urtica* spp.*	15
**2015**	2018	Landes pine tar**	17
**2016**		Onion oil*	17
**2012**		Talc E553b*	70
**2017**	2020	Cow milk*	30
**2017**		Grape (*Vitis vinifera*) cane tannins**	29
**2018**		L-cysteine*	22
**2016**		Propolis**	21
**2015**		*Saponaria* off roots**	37
**2019**	2021	*Allium cepa* bulb extract*	19
**2015**		Comfrey steeping**	26
**2019**		Dimethyl sulfide*	15
**2018**		Oleoresin capsicum**	17
**2018**		Willow stem and bark extract**	-
**2018**	2022	Chitosan*	27

* Approved Basic substances; ** Refused basic substances

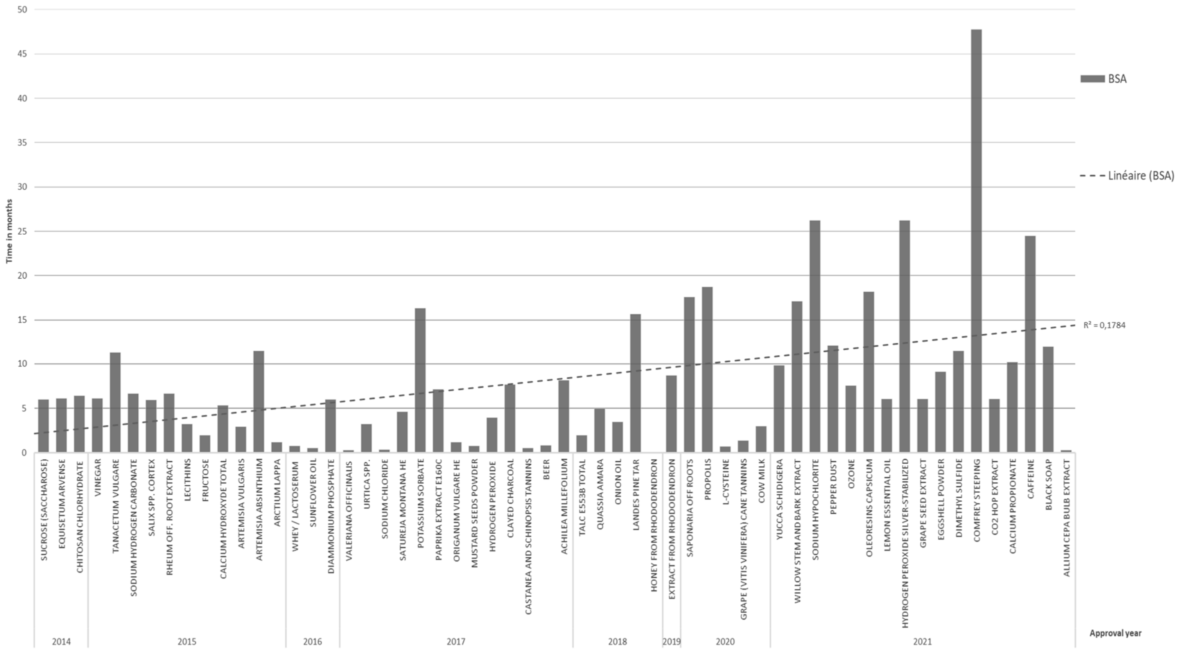

Figure S1. Time needed for Basic Substance Application admissibility evaluation over time (bars) and tendency line (dotted line).
